# Meropenem as a non-guideline antibiotic: the factor associated with mortality of mortality in *Listeria monocytogenes* meningitis

**DOI:** 10.3389/fpubh.2026.1895883

**Published:** 2026-10-12

**Authors:** Qiangsheng Feng, Yuejuan Song, Yuan Xing, Julin Zhan, Tuanjie Che, Xu Heng, Xiliang Bin, Xiaoqin Ha

**Affiliations:** 1 Department of Clinical Laboratory, The 940th Hospital of Joint Logistics Support Force of People’s Liberation Army, Lanzhou, China; 2 School of Public Health, Gansu University of Chinese Medicine, Lanzhou, China; 3 Innovation Center of Functional Genomics and Molecular Diagnostics Technology of Gansu Province, Lanzhou, China

**Keywords:** *Listeria monocytogenes*, meningitis, meropenem, mortality, risk factor

## Abstract

**Background:**

*Listeria monocytogenes* meningitis has high mortality. Evidence on meropenem’s real-world efficacy in Chinese adults is lacking.

**Methods:**

This retrospective study of 19 Listeria meningitis cases used CSF/blood culture and ROC analysis for diagnosis, and applied Fisher’s exact test to explore the association between meropenem treatment and mortality.

**Results:**

In 19 patients with *L. monocytogenes* meningitis (median age 56 years, male/female 2.16:1), *L. monocytogenes* was the second most common cause (70.3% of Listeria infections). Underlying diseases were present in 10 patients, and MRI showed periventricular changes (9 cases), bifrontal lesions (2), hydrocephalus (1), and normal findings (4). Of 10 CSF-positive patients, 2 had concurrent positive blood cultures; mean time to blood culture positivity was 19.3 ± 9.3 h. All tested isolates were susceptible to ampicillin, penicillin, TMP-SMX (Trimethoprim-sulfamethoxazole), and meropenem. Serum PCT (AUC = 0.980), CSF WBC (AUC = 1.000), and CSF protein (AUC = 0.959) showed excellent diagnostic performance. In exploratory univariate analysis, Fisher’s exact test identified non-guideline antibiotic administration as the factor associated with mortality of mortality (*p* = 0.035).

**Conclusion:**

Non-guideline antibiotic use (meropenem) was the factor associated with mortality of mortality in adult *L. monocytogenes* meningitis (40% mortality). First-line therapy should remain ampicillin/penicillin; meropenem should be reserved for alternative use only based on existing guidelines, not on the findings of this study alone.

## 1 Introduction

*Listeria monocytogenes* is one of the pathogens causing severe central nervous system infections. Although its annual incidence is relatively low (approximately 0.9–4.7 per million population globally), the disease is critical, progresses rapidly, and has a significantly higher mortality rate than acute bacterial meningitis caused by other pathogens. According to reports, the overall mortality rate of *L. monocytogenes* infection is 15–20%, with the case fatality rate in meningitis patients reaching as high as 33%, and the incidence of poor clinical prognosis (Glasgow Outcome Scale score <4) is approximately 47%, both significantly higher than other bacterial meningitides (*p* = 0.015 and 0.009, respectively) (1, 2). Affected patients often have underlying immunosuppressive conditions, such as advanced age, malignancies, diabetes, organ transplantation, or long-term corticosteroid use, which further complicates treatment decisions.

Currently, international authoritative guidelines such as those from ECCO Guidelines and Chinese guidelines clearly recommend ampicillin or penicillin combined with gentamicin as first-line therapy for *L. monocytogenes* meningitis, with a usual treatment duration of 3 weeks (3, 4). Because Listeria is intrinsically resistant to cephalosporins, the use of third-generation cephalosporins like ceftriaxone as empirical monotherapy has been shown to be inadequate empirical antibiotic therapy and is significantly associated with higher 30-day mortality. Although meropenem has some *in vitro* activity against Listeria and is listed as an alternative option in some guidelines, its real-world clinical efficacy remains controversial. A Danish multicenter retrospective study including 229 patients with invasive listeriosis (57 with meningitis) reported that patients receiving definitive therapy with benzylpenicillin or aminopenicillins had significantly lower 30-day mortality than those receiving meropenem (adjusted OR 0.3, 95% CI 0.1–0.8), suggesting that using meropenem as definitive therapy is associated with higher mortality (5). However, this evidence comes mainly from European populations, and real-world data from Chinese populations to verify the actual clinical benefit or risk of meropenem in this disease are lacking.

At present, Chinese data on whether meropenem, as a non-guideline antibiotic, independently affects patient prognosis in *L. monocytogenes* meningitis are extremely limited. Given the rarity of this disease, we designed this study as a descriptive case series to systematically evaluate the current use of meropenem and its relationship with in-hospital mortality by retrospectively analyzing the clinical data of 19 patients with *L. monocytogenes* meningitis from our hospital, with the aim of generating hypotheses for antibiotic selection and clinical decision-making for this disease in the Chinese population.

## 2 Materials and methods

### 2.1 Study design

The selection criteria is described (see Figure 1)

**Figure 1:**
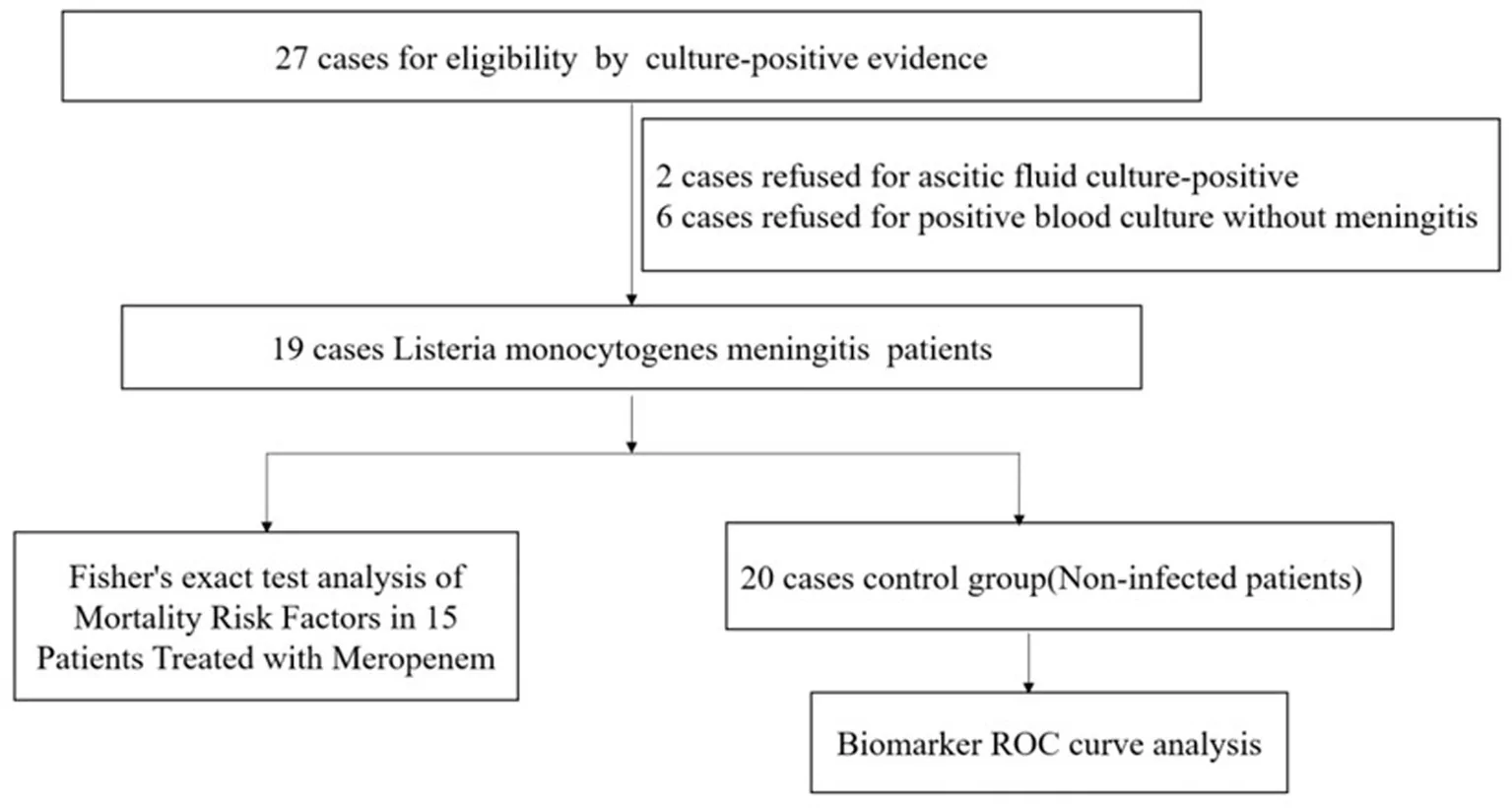
Selection criteria for the inclusion of patients with *Listeria monocytogenes* meningitis.

*Case identification*: Among 19 cases of *L. monocytogenes* meningitis, nine had positive CSF cultures with negative blood cultures, two had both CSF and blood cultures positive, and eight had positive blood cultures and were ultimately diagnosed with intracranial infection from 2012 to 2025. Patients with hematological diseases and agranulocytosis were not included in the analysis of WBC and NEU%. The control group had a mean age of 47 years (IQR: 29–67; range: 12–79 years) and a male-to-female ratio of 1.5:1. The most common clinical diagnoses in the control group were hematologic diseases and headache, each occurring in 5 patients (25% each), followed by dizziness and autoimmune encephalitis in 2 patients each (10% each). The remaining diagnoses—low back pain, intracranial aneurysm, peripheral neuropathy, type 2 diabetes mellitus, lymphoma, and cognitive impairment—each accounted for 6.25% (1 patient each). Confirmed case: positive CSF culture for *L. monocytogenes* (4). Severe comorbidity was defined as the presence of two or more chronic conditions, or any single condition requiring long-term immunosuppressive therapy (e.g., active malignancy, organ transplantation, or hematological disease.

### 2.2 Setting

This study conducted a retrospective review of clinical data from patients diagnosed with *L. monocytogenes* meningitis patients at the 940th Hospital of the Joint Logistics Support Force of the People’s Liberation Army. Statistical analyses were performed on factors such as antimicrobial therapy Meropenem (second-line therapy, 15 cases) vs. other non-guideline antibiotics (4 cases, patients who received cefoperazone/sulbactam (*n* = 2), imipenem (*n* = 1), or no antibiotics (*n* = 1), clinical characteristics, underlying diseases.

### 2.3 Ethical oversight

The study was approved by the Ethics Committee of the 940th Hospital of the Joint Logistics Support Force of the People’s Liberation Army (approval No. 2025KYLL21126, December 2025). The committee waived the need for informed consent. The study adhered to the ethical standards outlined in the Declaration of Helsinki (1975) and its amendments.

### 2.4 Participants

This study carefully selected participants from 2012 to 2025, ultimately including 19 cases eligible patients who were followed for 360-days.

### 2.5 Exposure variables

Demographic and clinical characteristics were analyzed as exposure variables. This included age, sex, use of antimicrobial agents, antimicrobial therapy (sensitive antibacterial drugs: Meropenem (second-line therapy) vs. other non-guideline antibiotics, ICU admission, Administration time, and underlying diseases. Patients who died within 24 h were included in the analysis, and their brief antibiotic exposure is noted as a potential source of bias in the Limitations.

### 2.6 Endpoint

The primary endpoint was in-hospital mortality among *L. monocytogenes* meningitis inpatients for in-hospital mortality.

### 2.7 *Listeria monocytogenes* culture, identification and antimicrobial susceptibility test

All the subjects recorded episodes who were hospitalized and suspected of BSIs between January 2012 and January 2025, blood cultures were obtained using BacT/ALERTblood culture bottles (bio-Mérieux, Inc., Durham, NC) or BD FA and SN blood culture bottles and incubated in the BacT/ALERT 3D (bioMérieux, Inc) or BD FX 400 automatic monitoring system for a week in the clinical microbiology laboratory of the hospital. When Bottles flagged as positive after Gram-positive bacilli, report the critical value and switch to blood culture onto blood tablet and Chinese blue agar plates and incubated at 35 °C CO_2_ for 24 h. Cerebrospinal fluid (CSF) specimens should be delivered to the laboratory immediately (<15 min). An aliquot of 2-3 mL of CSF is centrifuged at 2000–3000 rpm for 10 min, and the sediment is inoculated onto blood agar plates, chocolate agar plates, and China blue agar plates. The plates are incubated at 35 ± 1 °C in a 5–10% CO₂ atmosphere. After 24 h colony formation, microbial identification was performed using the corresponding GN card on the VITEK Compact-II automatic microorganism identification system or MALDI-TOF MS. Antimicrobial susceptibility testing for *L. monocytogenes* was performed using the E-test method. The test medium was Mueller-Hinton agar supplemented with 5% defibrinated horse blood and 20 mg/L β-NAD (MH-F), following the CLSI M45 guidelines.

### 2.8 Statistical analyses

Statistical analyses were performed using SPSS 22.0. Fisher’s exact test analysis of Mortality Risk Factors in 15 patients treated with meropenem (*p* < 0.05), due to the small sample size and limited number of events, multivariable analysis could not be performed, and thus all reported associations are unadjusted. Clinical biomarkers were assessed using receiver operating characteristic (ROC) curves. All statistical findings should be interpreted as descriptive and hypothesis-generating.

## 3 Results

This study analyzed 19 cases of *L. monocytogenes* meningitis diagnosed between 2013 and 2025. The median age was 56 years (IQR: 28–66; range: 17–89 years), with a male-to-female ratio of 2.16:1. *L. monocytogenes* accounted for 70.3% (19/27) of all Listeria infections in our hospital, making it the second most common causative pathogen after *Streptococcus pneumoniae*.

Among the 19 patients, 10 had underlying diseases, including 4 cases of hematologic diseases or malignancies and 3 cases of cerebrovascular diseases. Imaging was performed in 16 patients (all by MRI): periventricular demyelinating changes were observed in 9 cases, bifrontal hypodense lesions in 2, hydrocephalus in 1, and normal findings in 4.

Blood culture results showed an anaerobic/aerobic bottle ratio of 1:1, with a mean time to positivity of 19.3 ± 9.3 h. Of the 10 patients with positive cerebrospinal fluid (CSF) culture, two had concurrent positive blood cultures, while the remaining eight had negative blood cultures. Antimicrobial susceptibility testing was performed on six *L. monocytogenes* isolates using ampicillin, penicillin, trimethoprim-sulfamethoxazole, and meropenem; no resistant strains were detected (Table 1, Figure 2).

**Table 1:** *Listeria monocytogenes* meningitis susceptibility testing (*n* = 6 cases).

Cases	Ampicillin	Penicillin	Trimethoprim-sulfamethoxazole	Meropenem
	MIC breakpoint (≤1 mg/L)	MIC breakpoint (≤1 mg/L)	MIC breakpoint (≤0.06 mg/L)	MIC breakpoint (≤0.25 mg/L)
	Zone diameter breakpoint (≥16 mm)	Zone diameter breakpoint (≥13 mm)	Zone diameter breakpoint (≥29 mm)	Zone diameter breakpoint (≥26 mm)
Cases 1	S (25 mm)	S (0.25 mg/L)	S (0.016 mg/L)	S (0.064 mg/L)
Cases 2	S (16 mm)	S (13 mm)	S (0.032 mg/L)	S (0.125 mg/L)
Cases 3	S (24 mm)	S (0.5 mg/L)	S (30 mm)	S (0.125 mg/L)
Cases 4	S (26 mm)	S (1 mg/L)	S (0.032 mg/L)	S (0.064 mg/L)
Cases 5	S (26 mm)	S (16 mm)	S (29 mm)	S (28 mm)
Cases 6	S (25 mm)	S (1 mg/L)	S (30 mm)	S (28 mm)

**Figure 2:**
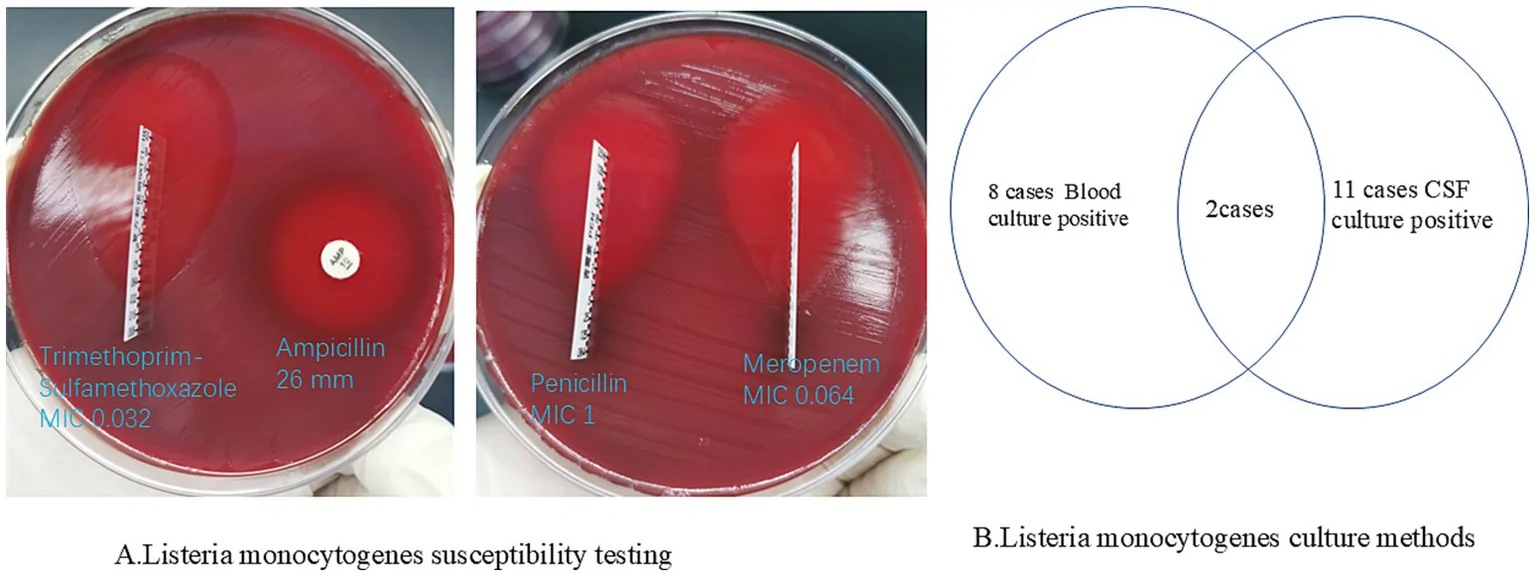
**(A)** *Listeria monocytogenes* susceptibility testing medium: Mueller-Hinton agar supplemented with 5% defibrinated horse blood and 20 mg/L β-NAD (MH-F) (MIC mg/L). **(B)** Of the 10 patients with positive cerebrospinal fluid (CSF) culture, two had concurrent positive blood cultures, while the remaining eight had negative blood cultures.

The diagnostic performance of selected biomarkers for *L. monocytogenes* meningitis was evaluated using receiver operating characteristic (ROC) curve analysis in the cohort of 19 patients (Figure 3, Table 2). Serum procalcitonin (PCT) demonstrated excellent diagnostic accuracy with an area under the curve (AUC) of 0.980. At the optimal cutoff of 0.096 ng/mL, PCT yielded a sensitivity of 0.929 and a specificity of 1.000. CSF white blood cell count (WBC) also showed perfect diagnostic performance (AUC = 1.000), with a sensitivity of 0.929 and a specificity of 1.000 at a cutoff of 79 × 10^6^/L. CSF protein had an AUC of 0.959, achieving a sensitivity of 0.929 and a specificity of 1.000 at a cutoff of 812.5 mg/L.

**Figure 3:**
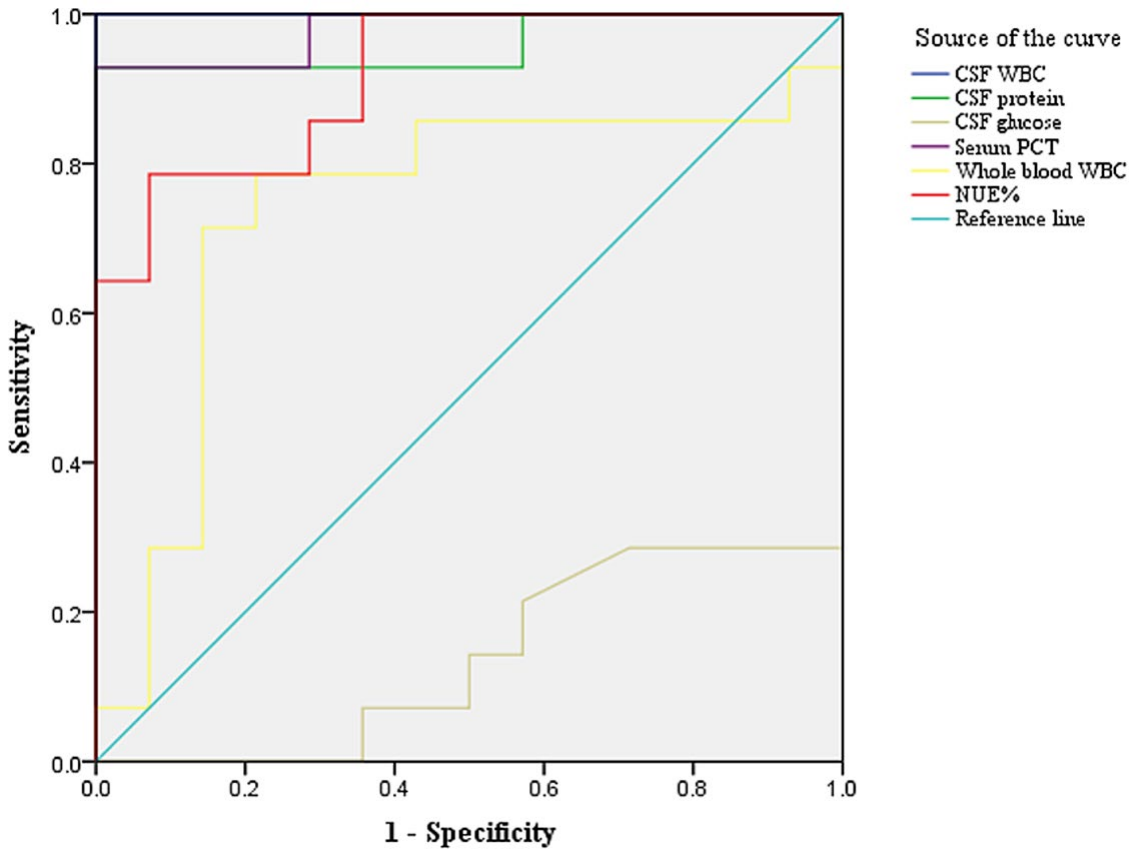
Diagnostic performance of biomarkers for distinguishing *Listeria monocytogenes* Meningitis (*n* = 19) from non-infected controls (*n* = 20) using ROC curve analysis. Serum procalcitonin (PCT) demonstrated excellent diagnostic accuracy with an area under the curve (AUC) of 0.980. At the optimal cutoff of 0.096 ng/mL, PCT yielded a sensitivity of 0.929 and a specificity of 1.000. CSF white blood cell count (WBC) also showed perfect diagnostic performance (AUC = 1.000), with a sensitivity of 0.929 and a specificity of 1.000 at a cutoff of 79 × 10^6^/L. CSF protein had an AUC of 0.959, achieving a sensitivity of 0.929 and a specificity of 1.000 at a cutoff of 812.5 mg/L.

**Table 2:** AUC for the biomarker diagnostic value in *Listeria monocytogenes* meningitis (*n* = 19 cases).

Biomarkers	AUC value	Cut-off value	Sensitivity	Specificity	95% confidence interval	
					Lower	Upper
CSF WBC (×10^6^/L)	1.000	79	0.929	1.000	1.000	1.000
CSF protein (mg/L)	0.959	812.5	0.929	1.000	0.881	1.000
CSF glucose (mmol/L)	0.862	2.2	1.000	0.714	0.721	1.000
Serum PCT (ng/ml)	0.980	0.096	0.929	1.000	0.925	1.000
WBC (×10^9^/L)	0.740	—	—	—	—	—
NEU% (%)	0.918	82.05	0.786	0.929	0.809	1.000

Biomarkers significance differences analysis *Listeria monocytogenes* meningitis (*n* = 19 case) vs. control (non-infected patients, 20 cases) using Mann–Whitney *U* test, the *p*-value (<0.001) for CSF WBC [95% CI, *Z* = 4.516, *p* < 0.001, [620 (292, 1197) vs. 3 (1, 12)], CSF protein [95% CI, *Z* = 4.135, *p* < 0.001, [1859.5 (1349.7, 2981.7) vs. 352.0 (332.0, 405.5)], CSF glucose () [95% CI, *Z* = 3.264, *p* = 0.001, [1.7 (0.3, 2.9) vs. 3.1 (2.7, 3.4)], Serum PCT [95% CI, *Z* = 4.320, *p* < 0.001, 0.45 (0.12, 1.71) vs. 0.06 (0.05, 0.08)], WBC (×10^9^/L) [95% CI, *Z* = 2.160, *p* = 0.031, 14.27 (9.76, 19.26) vs. 6.67 (6.22, 10.23)]. NEU% (%) [95% CI, *Z* = 3.768, *p* < 0.001, 86.5 (82.2, 92.3) vs. 73.3 (67.7, 81.5)]. PCT, procalcitonin; WBC, white blood cell; NEU%, neutrophil %.

In a cohort of 15/19 patients with *L. monocytogenes* meningitis treated with meropenem (a non-guideline antibiotic), the mean pre-admission symptom duration was 3 days (range 1–7 days). Meropenem was initiated within 24 h of admission in 13 patients (72%). The overall mortality rate was 40% (6/15): three deaths occurred within the first 24 h and three between days 3 and 13, with no deaths beyond day 13. Performed an exploratory analysis to examine the association between MRI findings (periventricular changes, hydrocephalus, and bifrontal lesions) and mortality. No statistically significant association was found (*p* > 0.05 for all comparisons). In exploratory univariate analysis, Fisher’s exact test identified non-guideline antibiotic administration as the factor associated with mortality of mortality (*p* = 0.035) (Table 3). Given the small sample size and unadjusted nature of this analysis, this finding should be interpreted with caution and is hypothesis-generating rather than confirmatory.

**Table 3:** *Listeria monocytogenes* meningitis meropenem administration survival rate (*n* = 19cases).

Demographics and clinical characteristics	Total (*n* = 15 cases)	Non-survivor (*n* = 6 cases)	Survivor (*n* = 9 cases)	*p*
Sex				*p* = 0.404
Female	11 (73%)	5 (45%)	6 (55%)	
Male	4 (27%)	1 (25%)	3 (75%)	
Age (IQR)				*p* = 0.833
≥53	8 (53%)	3 (37%)	5 (63%)	
<53	7 (47%)	3 (43%)	4 (57%)	
Pre-admission duration of illness(days)				*p* = 0.622
≥3	9 (53%)	3 (33%)	6 (66%)	
<3	6 (47%)	3 (50%)	3 (50%)	
Imaging changes				*p* = 0.530
Yes	9 (69%)	2 (22%)	7 (78%)	
No	4 (31%)	2 (50%)	2 (50%)	
Severe comorbidity				*p* = 0.608
Yes	7 (47%)	2 (29%)	5 (71%)	
No	8 (53%)	4 (50%)	4 (50%)	
Time to susceptible antimicrobial therapy				*p* = 0.532
≤24 h	13 (72%)	8 (61%)	5 (39%)	
>24 h	5 (28%)	1 (20%)	4 (80%)	
Antimicrobial agents				*p* = 0.035
Meropenem (second-line therapy)	15 (79%)	6 (40%)	11 (60%)	
Non-guideline antibiotics/without antibiotic use	4 (21%)	4 (100%)	0	
Monocytogenes meningitis				*p* = 0.236
Yes	19 (70%)	10 (53%)	9 (47%)	
No	8 (30%)	2 (25%)	6 (75%)	
CSF protein				*p* = 0.266
≥1859	7 (50%)	4 (57%)	3 (43%)	
<1859	7 (50%)	1 (14%)	6 (86%)	
CSF WBC				*p* = 0.580
≥620	6 (40%)	3 (41%)	3 (59%)	
<620	8 (60%)	2 (20%)	6 (80%)	
CSF glucose				*p* = 1.000
≤1.7	6 (43%)	2 (33%)	4 (66%)	
>1.7	8 (57%)	3 (37%)	5 (63%)	

Meropenem: according to authoritative guidelines such as IDSA and WHO, the recommended second-line therapy is meropenem. Non-guideline therapy: cefoperazone/sulbactam (2 cases), imipenem (1 case), without antibiotic use (1 case). Data are median (IQR) or *n* (%). *p*-values were calculated by Fisher’s exact test, as appropriate. Severe comorbidities were present in 7 cases, including 3 cases of cerebral infarction, 2 cases of hematological diseases, 1 case of uremia, and 1 case of Sjögren’s syndrome.

## 4 Discussion

This retrospective study of 19 adult cases of *L. monocytogenes* meningitis (2013–2025) provides a detailed characterization of the clinical, radiological, microbiological, and biomarker profiles of this severe infection. As a descriptive case series, the most striking finding was a 40% mortality rate among the 15 patients treated with meropenem, a non-guideline antibiotic, and the identification of non-guideline antibiotic administration as the factor associated with mortality of death (*p* = 0.035, Fisher’s exact test). However, due to the small sample size and lack of multivariable adjustment, this should be interpreted as an exploratory finding that requires validation in larger cohorts. Below we discuss the key observations in the context of the current literature.

The median age of 56 years, male predominance (2.16:1), and high prevalence of underlying diseases (10/19, especially hematological malignancies and cerebrovascular disorders) are consistent with the well-known predilection of *L. monocytogenes* for immunocompromised and older adult(s) individuals (6, 7). In our hospital, *L. monocytogenes* accounted for 70.3% of all listeriosis cases and was the second most common pathogen (after *Streptococcus pneumoniae*) causing bacterial meningitis. This underscores the importance of considering Listeria in the differential diagnosis of adult community-acquired meningitis, particularly when the presentation is subacute, when parenchymal brain signs are present, or when the patient has an underlying immunodeficiency (8).

Magnetic resonance imaging revealed periventricular demyelinating changes in 9/16 patients and bifrontal hypodense lesions in 2/16, which aligns with the known propensity of Listeria to cause rhombencephalitis and subependymal infiltration (9). Blood cultures had a mean time to positivity of 19.3 ± 9.3 h with an equal anaerobic/aerobic bottle ratio, but only 2 of the 10 CSF culture-positive patients had concurrent positive blood cultures. This indicates that a negative blood culture does not rule out Listeria meningitis, a finding previously noted in large prospective cohorts (10). All six tested isolates were susceptible to ampicillin, penicillin, trimethoprim-sulfamethoxazole, and meropenem, excluding resistance as a cause of the poor outcome in the meropenem-treated group.

ROC analysis showed that serum procalcitonin (PCT) had an AUC of 0.980, with an optimal cut-off of 0.096 ng/mL (sensitivity 92.9%, specificity 100%). This threshold is considerably lower than the classic cut-off used for typical purulent meningitis (e.g., 0.5 ng/mL) (11), reinforcing the concept that Listeria meningitis induces a relatively mild systemic inflammatory response. CSF white blood cell count (AUC 1.000; cut-off 79 × 10^6^/L) and CSF protein (AUC 0.959; cut-off 812.5 mg/L) also exhibited near-perfect diagnostic accuracy. The remarkably high CSF protein cut-off likely reflects severe disruption of the blood–brain barrier and intense fibrin exudation, a characteristic pathological feature of listerial infection (12). Combined use of these three biomarkers may enable early recognition and prompt empiric anti-Listeria therapy. However, the proposed cutoffs for PCT, CSF WBC, and CSF protein—derived from the same cohort—may be subject to overfitting and require external validation prior to clinical use.

In the present cohort of 15 adult patients with *L. monocytogenes* meningitis who received meropenem (a non-guideline antibiotic), the overall mortality was 40% (6/15). Thirteen patients (72%) received meropenem within 24 h of admission; nevertheless, six deaths occurred, three of them within the first 24 h (half of all fatal cases). Fisher’s exact test identified non-guideline antibiotic administration as the factor associated with mortality of mortality (*p* = 0.035). These findings align closely with the Danish nationwide cohort study by Thønnings et al. (5), which included 229 patients with invasive listeriosis (57 with meningitis) and demonstrated that definitive therapy with meropenem was associated with a significantly higher 30-day mortality compared with benzylpenicillin or aminopenicillins (adjusted OR = 0.3, 95% CI 0.1–0.8). That study concluded that both inadequate empirical antibiotic therapy and definitive therapy with meropenem were independently associated with significantly higher 30-day mortality. Our single-center experience provides independent, real-world evidence that corroborates this finding, reinforcing the notion that in adult *L. monocytogenes* meningitis, meropenem should not be used as first-line definitive therapy. Our single-center experience provides independent, real-world evidence that corroborates this finding, reinforcing the notion that in adult *L. monocytogenes* meningitis, meropenem should not be used as first-line definitive therapy. However, we emphasize that our findings are descriptive and hypothesis-generating, and should not be interpreted as causal.

The contrast with pediatric data is notable (Table 4). In a 10-year retrospective study from Chongqing Children’s Hospital (20 children), meropenem was used in 75% (15/20) of patients, always together with ampicillin, and all 12 children with long-term follow-up had favorable outcomes (13). Even more strikingly, a study from Beijing Children’s Hospital (41 non-neonatal children) reported that 97.5% of the children received meropenem (alone or in combination), and every child achieved a Glasgow Outcome Scale score of 5 (good prognosis), with no deaths (14). Thus, while meropenem appears to be safe and effective in pediatric *L. monocytogenes* meningitis, the same antibiotic is associated with higher mortality in adults. However, it is critical to note that these pediatric data originate from different cohorts with different study designs, age groups, baseline immune status, and outcome definitions. Direct cross-population comparisons are therefore limited, and caution is warranted when contrasting pediatric and adult findings. In the present adult cohort, meropenem use was associated with higher mortality. This association should not be interpreted as a direct harmful effect of the drug without considering confounding by indication. Patients who received meropenem may have had more severe underlying disease (e.g., delayed diagnosis, treatment failure on initial therapy, or multi-organ dysfunction), which itself could explain the observed higher mortality. Several factors may contribute to the divergent associations between adults and children observed association in adults, including: (i) host immune status—53% of our adult patients had immunosuppressive comorbidities (diabetes, malignancies, organ transplantation, long-term corticosteroid use), whereas children are overwhelmingly immunocompetent; (ii) disease severity and diagnostic delay—adults often present with more advanced illness and multiple organ dysfunction; and (iii) pharmacokinetic differences—meropenem clearance is more rapid in children, potentially reducing drug-related risks, though this remains speculative. Rather than concluding that but safe in children, we emphasize that the current data support an association in adults that may be driven by confounding factors, and that pediatric evidence should not be directly extrapolated to adult populations.

**Table 4:** Pediatric cohort evidence: meropenem safe and effective, even preferred.

Source	Meropenem use	Main findings	Effect on mortality
Danish nationwide study (2016) (5)	28 definitive therapy	Significantly higher mortality vs. benzylpenicillin/ampicillin	OR = 0.3 (0.1–0.8)
Chongqing Children’s Hospital (2024) (13)	15/20 (75%)	Meropenem and ampicillin preferred; good outcomes	All followed patients had good outcomes
Beijing Children’s Hospital (2025) (14)	41/41 (100%)	Meropenem as alternative/combination; all good outcomes	No deaths
Multiple case reports (2, 15–19)	Combination therapy	Effective	Survived

Current IDSA and ESCMID guidelines consistently recommend ampicillin or penicillin combined with gentamicin as first-line therapy for *L. monocytogenes* meningitis, with a usual treatment duration of 3 weeks. Meropenem is considered only as an alternative when first-line agents are contraindicated (e.g., severe β-lactam allergy) or have failed. Our data support this hierarchical approach for adults. For children, the published Chinese pediatric series suggest that meropenem can be used effectively as part of combination therapy or even as a preferred regimen, but this should be interpreted with caution given the absence of large prospective trials.

## 5 Limitation

The main limitations of our study are its small sample size (15 patients) and single-center retrospective design, which may introduce selection bias. The mortality estimate of 40% should be interpreted with caution, and larger multi-center prospective studies are needed to confirm our findings. Nevertheless, the consistency with the Danish nationwide study supports the external validity of our results. Further research should focus on identifying host factors that predict poor response to meropenem in adults and on optimizing treatment strategies in different age groups. However, the proposed cutoffs for PCT, CSF WBC, and CSF protein—derived from the same cohort—may be subject to overfitting and require external validation prior to clinical use.

## Data Availability

The original contributions presented in the study are included in the article/supplementary material, further inquiries can be directed to the corresponding author.
